# ^222^Rn and ^226^Ra Concentrations in Spring Water and Their Dose Assessment Due to Ingestion Intake

**DOI:** 10.3390/ijerph19031758

**Published:** 2022-02-03

**Authors:** Ryohei Yamada, Masahiro Hosoda, Tomomi Tabe, Yuki Tamakuma, Takahito Suzuki, Kevin Kelleher, Takakiyo Tsujiguchi, Yoshiki Tateyama, Eka Djatnika Nugraha, Anna Okano, Yuki Narumi, Chutima Kranrod, Hirofumi Tazoe, Kazuki Iwaoka, Yumi Yasuoka, Naofumi Akata, Tetsuya Sanada, Shinji Tokonami

**Affiliations:** 1 Graduate School of Health Sciences, Hirosaki University, 66-1 Honcho, Hirosaki, Aomori 036-8564, Japan; h20gg704@hirosaki-u.ac.jp (R.Y.); h19gg703@hirosaki-u.ac.jp (Y.T.); suzuki-takahito@fujielectric.com (T.S.); r.tsuji@hirosaki-u.ac.jp (T.T.); h20gg701@hirosaki-u.ac.jp (E.D.N.); 2 Institute of Radiation Emergency Medicine, Hirosaki University, Hirosaki, Aomori 036-8564, Japan; K.Kelleher@epa.ie (K.K.); kranrodc@hirosaki-u.ac.jp (C.K.); tazoe@hirosaki-u.ac.jp (H.T.); akata@hirosaki-u.ac.jp (N.A.); tokonami@hirosaki-u.ac.jp (S.T.); 3 School of Health Sciences, Hirosaki University, Hirosaki, Aomori 036-8564, Japan; tomomi.mpa@gmail.com (T.T.); y.ateyama07@gmail.com (Y.T.); anna.okano271828@gmail.com (A.O.); n.trapezohedron@outlook.jp (Y.N.); 4 Office of Radiation Protection and Environmental Monitoring, Environmental Protection Agency, Clonskeagh Square, D14 H424 Dublin, Ireland; 5 National Institute of Radiological Sciences, National Institutes for Quantum Science and Technology, 4-9-1 Anagawa, Inage, Chiba 263-0024, Japan; iwaoka.kazuki@qst.go.jp; 6 Radioisotope Research Center, Kobe Pharmaceutical University, Kobe, Hyogo 658–8558, Japan; yasuoka@kobepharma-u.ac.jp; 7 Department of Radiological Technology, Faculty of Health Sciences, Hokkaido University of Science, Sapporo 006-8585, Japan; sanada-t@hus.ac.jp

**Keywords:** ^222^Rn, ^226^Ra, spring water, activity concentration, dose assessment

## Abstract

^222^Rn and ^226^Ra concentrations of less than a few to several thousands of Bq L^−^^1^ have been observed in several underground bodies of water around the world. Although regulations for these concentrations in water have been implemented internationally, there are currently no regulations in place in Japan. However, concentrations that exceed these internationally recognized regulatory values have also been observed in Japan. In this study, concentrations in spring water in the northern part of Japan were measured and the effective dose from intake of the water was evaluated. ^222^Rn concentrations were measured using a liquid scintillation counter, and ^226^Ra concentrations were measured using a high purity germanium detector after chemical preparation. The measured ^222^Rn concentrations (=12.7 ± 6.1 Bq L^−1^) and ^226^Ra concentrations (<0.019–0.022 Bq L^−1^) did not exceed the reference values set by international and European/American organizations. A conservative estimate of the annual effective ingestion dose of 8 μSv for ^222^Rn and ^226^Ra obtained in this study is much smaller than the estimated overall annual effective dose of 2.2 mSv from natural radiation to the Japanese population. However, this dosage accounts for 8% of the WHO individual dosing criteria of 0.1 mSv/year for drinking water.

## 1. Introduction

It is well known that ^222^Rn, a decay product of ^226^Ra, is the second leading cause of lung cancer after tobacco smoking [1]. Since ^226^Ra and ^222^Rn are water soluble, groundwaters may contain ^226^Ra and ^222^Rn. The sources of ^226^Ra to the groundwater-phase result from the decay of dissolved ^230^Th, the dissolution of ^226^Ra-containing rocks, α-recoil associated with the α-decay of ^230^Th located in mineral surface layers, and the desorption reaction of ^226^Ra at the rock–water boundary [2]. The main source of ^222^Rn in water is due to α-recoil associated with the α-decay of ^226^Ra in the aquifer and dissolution of ^222^Rn generated in rocks [3]. In fact, ^222^Rn and ^226^Ra concentrations of less than a few to several thousands of Bq L^−1^ have been observed in several underground bodies of water around the world [4,5,6,7,8]. When these underground waters are used as drinking water, exposure due to ingestion is considered. In addition, if these waters are for domestic use, exposure from inhalation is also considered due to ^222^Rn released from the water into indoor air. Moreover, ^226^Ra is designated as a carcinogen (Group 1) in the International Agency for Research on Cancer classification [9], and its dose coefficient for the intake by the International Commission on Radiological Protection is relatively higher than that of other radionuclides. It is, therefore, important to measure ^222^Rn concentrations and ^226^Ra concentrations in underground water in the context of radiation protection of the public.

Under these circumstances, the United States Environment Protection Agency (USEPA) has established a regulatory value for ^222^Rn concentration in water. The first regulatory value (i.e., maximum contaminant level (MCL)) was proposed in 1991, and the value was 11 Bq L^−1^ [10]. The USEPA then conducted a further study in conjunction with the National Academy of Sciences, and as a result, the National Research Council (NRC) published a book entitled *Risk Assessment of Radon in Drinking Water* [11] in 1999. This study proposed 148 Bq L^−1^ as an Alternative MCL (AMCL), and this value is now used along with the MCL [12]. In addition, both the World Health Organization (WHO) recommend a guidance level [13] and European Union (EU) [14] recommend a parametric value of 100 Bq L^−1^ for ^222^Rn concentrations in drinking water. The USEPA [15] proposes 0.185 Bq L^−1^ as the MCL for ^226^Ra, whereas the WHO [13] recommends 1 Bq L^−1^ as the guidance level for ^226^Ra.

Thus, although regulations for ^222^Rn concentration and ^226^Ra concentrations in water have been implemented internationally, there are no regulations in place in Japan. However, ^222^Rn and ^226^Ra concentrations that exceed the international levels outlined above have also been observed in Japan [3,16,17]. It is, therefore, important to measure these concentrations in underground water, especially if it is used as drinking water, and estimate the effective dose for intake. However, only a limited number of these evaluations have been conducted in the past. In addition, the accumulation of measurement data may lead to the introduction of regulations in Japan in the future. In this study, ^222^Rn and ^226^Ra concentrations of spring water in Hirosaki City, where radioactivity in drinking water and dose assessment has not yet been carried out, was measured, and the effective dose from ingestion of the water was evaluated. There are about 20 sites where spring water can be collected in Hirosaki City, and although the chemistry of some spring waters is evaluated by administrative organizations, no analysis of radioactivity or evaluation of the effective dose due to ingestion has been conducted.

## 2. Materials and Methods

### 2.1. Water Sampling

**Figure 1:**
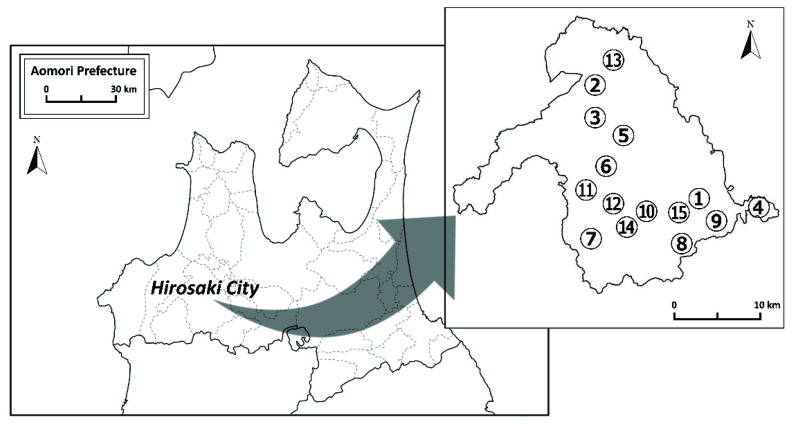
Locations of the sampling sites in Hirosaki City, Aomori Prefecture.

Spring water samples were collected at 15 locations in Hirosaki City, Aomori Prefecture (Figure 1). Hirosaki City (40°28′ N–40°45′ N, 140°09′ E–140°36′ E) is located in the southwestern part of Aomori Prefecture, which is located in the northern part of Honshu Island in the Japanese archipelago. The 15 sampling sites were selected from the spring waters that are known drinking water supplies and where sampling was possible. The basement geology of the sampling sites is shown in Table 1 [18]. According to Nemoto and Ujiie [18], the bedrock in Aomori Prefecture that includes the sampling sites are mainly composed of Jurassic accretionary complex and plutonic rocks of Cretaceous, which is penetrated Jurassic accretionary complex. This bedrock is covered in parts by a Neogene system and is also covered by sediments of post-Pleistocene and volcanic products. The aquifers at the sampling sites are located in Neogene Pliocene to Quaternary Pleistocene strata [19]. The basement geology and stratigraphic succession of Hirosaki City are described and shown in detail in the reports of Nemoto and Ujiie [18], Kogawa [19], and the National Institute of Advanced Industrial Science and Technology [20].

In this study, spring water samples were collected in 100 mL containers for ^222^Rn measurement, and approximately 10 L was collected in polyethylene containers for ^226^Ra measurement. One 100 mL and one 10 L sample from each of the sites were collected between August 2016 and September 2016. At Site No. 15, which is selected as one of “Meisui-100-sen” (100 best bodies of water) by the Ministry of the Environment, Japan [21], additional spring water samples were collected in 100 mL containers once a week from June 2016 to June 2017 to observe whether there existed any seasonal variation of radon concentrations. In addition, the pH, electric conductivity (EC), water temperature of the spring water samples, and atmospheric temperatures were measured. The pH, EC, and temperatures were measured using a pH meter (AS-711, HORIBA, Kyoto, Japan), an EC meter (B-771, HORIBA, Kyoto, Japan), and a thermometer (CT-220, CUSTOM Corporation, Tokyo, Japan), respectively. Moreover, the collected spring water was measured directly for gamma rays using a p-type high purity germanium (HPGe) detector (GEM30P4-70, ORTEC, Oak Ridge, USA), in order to confirm whether ^134^Cs and ^137^Cs, which was released as a result of the nuclear accident at the Fukushima Daiichi Nuclear Power Station (FDNPS), was observed.

### 2.2. ^222^Rn in Water Measurement

A total of 10 mL of sample was placed in a 20 mL glass vial containing a 10 mL liquid scintillator (High-Efficiency Mineral Oil Scintillator, PerkinElmer, Inc., Waltham, USA). The vial was shaken for 30 s and measured using a portable liquid scintillation counter (Triathler LSC, HIDEX, Turku, Finland) for 60 min at Hirosaki University, after leaving it for more than 4 h in a light-shielded area. Three samples were prepared for ^222^Rn analysis for each of the spring water sampling locations. ^222^Rn concentrations, $C_{\mathrm{Rn}}$ (Bq L^−1^), were evaluated using the following equation [22]:

(1) $$C_{\mathrm{Rn}}=\left(A_{0}-B_{0}\right)\times \exp\left(\frac{0.693\times t_{e}}{T}\right)\times \frac{1}{f}\times \frac{1}{V}\times CF$$

where $A_{0}$ is the integral counting rate of the sample (cps), $B_{0}$ is the integral counting rate of the background sample (cps), $t_{e}$ is the elapsed period from sampling (days), T is the half-life of ^222^Rn (=3.824 days), f is the sensitivity of the Triathler based on the previous report (=4.5 cps Bq^−1^) [22], V is the sampling volume (=10^−2^ L), and CF is calibration factor of the Triathler. $A_{0}$ and $B_{0}$ were calculated by the integral counting method [22,23,24], which is a method to evaluate the ^222^Rn concentration based on the integral counting rates of three-channel windows (50–1000 ch, 75–1000 ch, and 100–1000 ch). CF was evaluated by an interlaboratory intercomparison. For proper evaluation of ^222^Rn concentrations in water, an intercomparison was carried out between Hirosaki University and the Office of Radiation Protection and Environmental Monitoring, Environmental Protection Agency, Ireland (EPA-ORM). EPA–ORM is a radon-in-water measurement technique accredited to ISO 17025:2005 [25]. The water chosen for the intercomparison was a private domestic groundwater supply located in the southeast of Ireland, with a ^222^Rn concentration of approximately 750 Bq L^−1^, and five samples were prepared and measured. The arithmetic mean (± uncertainty (*k =* 1)) of the radon in water measurements analyzed by EPA–ORM was 765 ± 24 Bq L^−1^, and arithmetic mean (± uncertainty (*k* = 1)) for the measurements by Hirosaki University was 748 ± 8 Bq L^−1^. Therefore, the calibration factor was evaluated to be 1.02 ± 0.03 (uncertainty; *k* = 1), and this value was used as the CF in Equation (1).

### 2.3. ^226^Ra in Water Measurement

The chemical preparation of spring water samples was carried out according to an EPA–ORM test procedure, which is a barium sulfate (BaSO_4_) coprecipitation method and summarized by Hosoda et al. [26]. In this study, the sampling volume was 4 L. The precipitate was collected on a glass microfiber filter (Whatman GF/C 47 mmφ, Cytiva, Tokyo, Japan). The precipitate on the filter was dried overnight to prepare the sample for measurement. A sample was prepared for each spring water sample. The yield of barium was calculated by the gravimetric method according to Hosoda et al. [26].

The filter sample was placed with the precipitate side down in the measuring container. This measurement sample was sealed and stored for more than 30 days in order to establish radioactive equilibrium between ^226^Ra and its decay products. After preservation, gamma rays from samples were measured using a p-type HPGe detector (GEM-40190, ORTEC, Oak Ridge, USA), which was calibrated by a commercially available mixed activity gamma standard source (MX033U8PP, Japan Radioisotope Association, Tokyo, Japan). The measurement time was set as 80,000 s. The ^226^Ra activity was determined from the activity of ^214^Bi (609 keV).

### 2.4. Dose Assessment

The annual effective dose for ^222^Rn and ^226^Ra was estimated from the following equation, assuming that the spring water is consumed daily as drinking water [27,28]:

(2) $$D=C_{i}\times W\times K_{W}$$

where D is the annual effective dose due to ingestion (μSv), $C_{i}$ is the radioactive concentration (Bq L^−1^), W is the annual consumption (L), $K_{W}$ is the dose coefficient (μSv Bq^−1^) of ^222^Rn or ^226^Ra (6.9 × 10^−4^ μSv Bq^−1^ for ^222^Rn and 1.3 × 10^−1^ μSv Bq^−1^ for ^226^Ra [29]). According to the WHO [13], the annual ingested volume of drinking water is assumed to be 730 L y^−1^, which is equivalent to the standard WHO drinking water consumption rate of 2 L day^−1^. In this study, this value was used as the annual consumption, W, in Equation (2), which is similar to other previous studies [6,7].

## 3. Results

### 3.1. Water Quality and Radioactive Concentration of Sampling Water

The results of measuring pH, EC, water temperatures of spring water samples, the atmospheric temperature, ^222^Rn concentrations, and ^226^Ra concentrations for the 15 sampling sites are shown in Table 2. The ^222^Rn concentrations were in the range of 5.3–26.7 Bq L^−1^ with an arithmetic mean (± standard deviation (SD)) of 12.7 ± 6.1 Bq L^−1^. Although ^226^Ra concentrations were below the minimum detectable concentrations (MDCs) at many sites (12 of the 15 sites),values were observed at the other three sites, which ranged from 0.0093 to 0.022 Bq L^−1^. It should be noted that no radioactive cesium due to the FDNPS accident was observed from the gamma-ray measurements of the samples.

**Figure 2:**
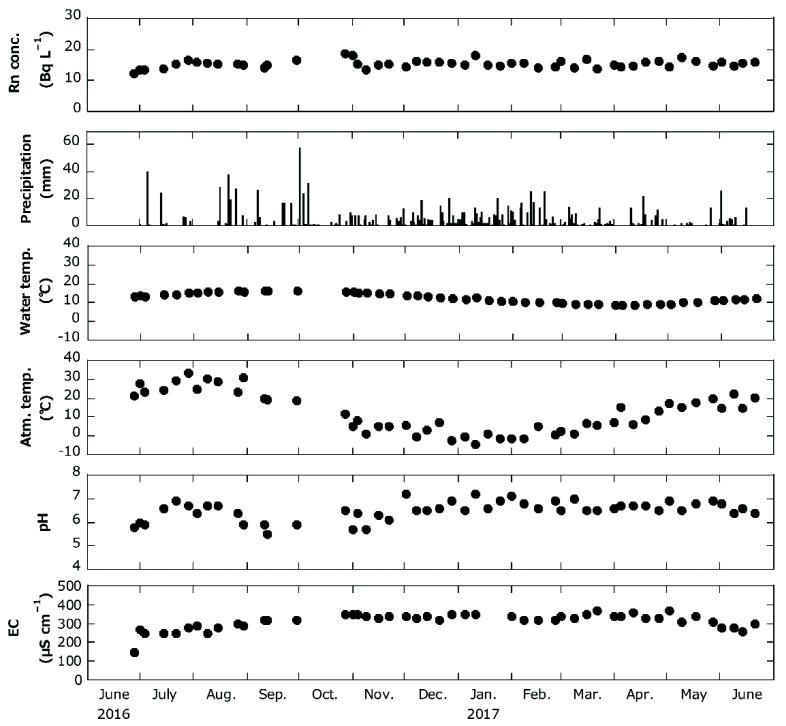
Periodic measurement results at Site No. 15.

The results of the periodic measurements of pH, EC, water temperatures of spring water samples, atmospheric temperature, and ^222^Rn concentration at Site No. 15 are shown in Figure 2. The range of variation for each parameter is shown in Table 2.

### 3.2. Dose Assessment Due to Ingestion

Table 3 shows the annual effective ingestion dose for ^222^Rn and ^226^Ra estimated using Equation (2), assuming that the spring water is consumed daily as drinking water (=730 L y^−1^). The annual effective ingestion dose for ^222^Rn fluctuated in the range of 3–13 μSv with an arithmetic mean (± SD) of 6 ± 3 μSv. For ^226^Ra the dose fluctuated in the range of <1–2 μSv. The annual effective ingestion dose for ^222^Rn and ^226^Ra fluctuated in the range of <4–14 μSv. If the ^226^Ra concentrations below the MDC level are considered to be the same concentrations as MDCs, the arithmetic mean of the annual effective ingestion dose is 8 μSv.

## 4. Discussion

The average of ^222^Rn concentrations in the 15 sampling sites and the results of those in water obtained elsewhere in Japan and around the world are shown in Table 4. The results of ^222^Rn concentrations in this study were in good agreement with the measurement results of the 35 sites of the “Meisui-100-sen” (100 best bodies of water) reported by Ishii [30]. In addition, the measured ^222^Rn concentrations did not exceed the AMCL of USEPA (=148 Bq L^−1^) or the levels indicated by the WHO and EU (=100 Bq L^−1^), as reported in some previous studies [5,31,32,33,34]. On the other hand, there were seven sampling sites that exceeded the MCL of USEPA (=11 Bq L^−1^). Therefore, if Japan introduces regulations on ^222^Rn concentrations in water, it will be necessary to carefully consider the regulatory values.

In order to investigate the relationship between the basement geology (Table 1) and ^222^Rn concentrations, ^222^Rn concentrations were classified based on the basement geology around the water sampling sites, and statistical processing was performed using EZR [36]. As a result of Shapiro–Wilk tests, normality was observed (*p* = 0.800) in ^222^Rn concentration in spring water for each basement geology. Therefore, although a one-way analysis of variance was performed, no significant difference was found for all basement geologies. It is well known that granitic rocks have high contents of natural radionuclides [37,38], and it has been reported that absorbed dose rates [39,40,41] and ^222^Rn concentrations in water [7] are elevated at sites where the basement geology is granite. According to Nemoto and Ujiie [18], however, the plutonic rocks consisting of granite were not included in the basement geology around the water sampling sites in this study. Therefore, it is considered that the difference in classified ^222^Rn concentrations could not be significantly confirmed.

**Figure 3:**
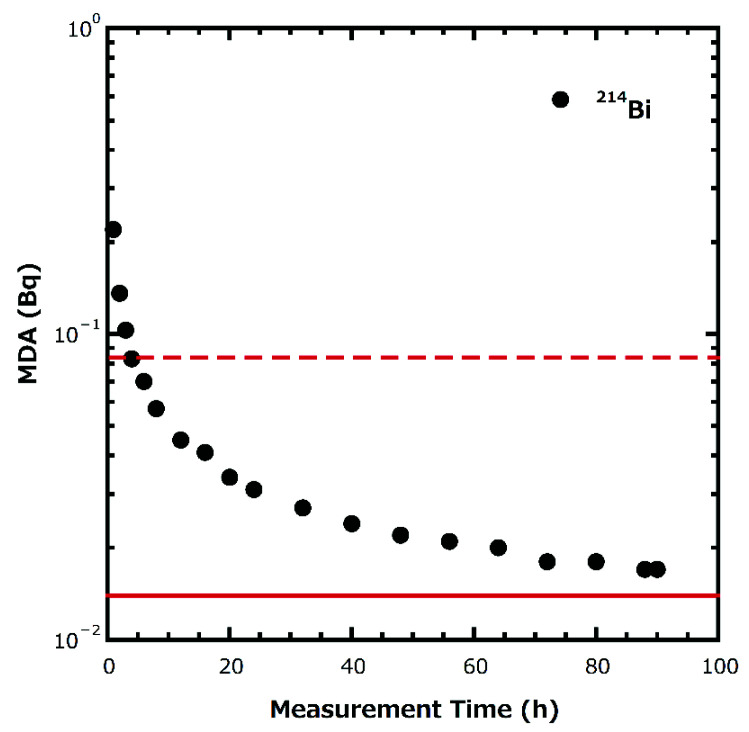
Relationship between measurement time and MDA. The red dashed line in the figure shows the radioactivity of the sample (=0.084 Bq L^−1^) when the sampling volume was 24 L, and the red line shows the radioactivity of the sample (=0.014 Bq L^−1^) when the sampling volume was 4 L. The measurement time in this study was 80,000 s (=22.2 h).

Although ^226^Ra concentrations were less than MDC at 12 sites, no concentration above the MCL of USEPA (=0.185 Bq L^−1^) or the guidance level of the WHO (= 1 Bq L^−1^) were identified at the remaining three sites where detectable values were observed. Further investigation was conducted on the MDC and an examination of the amounts of sampling volume and measurement time would be required for the future detection of ^226^Ra concentrations using this method was made. Figure 3 shows the results of minimum detectable activity (MDA) of ^214^Bi for long-term (maximum 90 h) gamma-ray measurement of a 24 L water sample containing 0.0035 Bq L^−1^ of ^226^Ra using the same chemical preparation as in this study. As shown in Figure 3, when the sampling volume was 24 L, ^214^Bi could be sufficiency quantified within 24 h of measurement. However, if the sampling volume is 4 L, a measurable activity concentration will not be achieved, even after 90 h of measurement (Figure 3). Therefore, it is necessary to increase the sampling volume in order to reliably determine ^226^Ra at the sampling sites in this study. In this case, extending the measurement time will have little influence on the limits of detection.

The ^222^Rn concentrations in spring water in this study were several orders of magnitudes higher than that of ^226^Ra, which has also been observed in previous studies [7,33,42]. Therefore, the source of ^222^Rn in water is not a result of the decay of dissolved ^226^Ra. In addition, according to Tricca et al. [43], weathering of rocks is not a significant source of ^222^Rn since this requires a high weathering rate. Some previous studies have reported that the occurrence of ^222^Rn is primarily controlled by α-recoil of ^222^Rn from the rock balanced by its decay [43,44,45]. Since it has recently been reported that grain size, distribution of Uranium in the rocks, and geological factors (e.g., faults and fracturing) of the aquifer are also important for the generation of ^222^Rn in water [45,46,47], obtaining this information would lead to a more detailed discussion of the sources of ^222^Rn in this study. Furthermore, it is also important to measure the chemical composition of the rock and water, as ^226^Ra can be removed by adsorption reactions such as ion exchange at the rock–water boundary and coprecipitation reactions resulting in deposition of sulfate, etc. [2].

The results of periodic measurement at Site No. 15 (Figure 2) indicate no significant seasonal variation in ^222^Rn concentration throughout the year, although some previous studies [48,49] have reported seasonal variations in ^222^Rn concentration in water. The atmospheric temperature at the sampling site fluctuated over time (Ave. ± SD = 12.3 ± 10.6 °C with a coefficient of variation (CV) of 86%); however, no significant change in water temperature was observed (Ave. ± SD = 12.6 ± 2.6 °C with a CV of 20%). According to Kogawa [19], since the depth of aquifers in Hirosaki City are tens of meters to ~200 m, the water temperature was considered to be less affected by large atmospheric temperature fluctuations. In addition, although there were periods of high precipitation, the ^222^Rn concentration in the water remained almost constant without any dilution. Moreover, there is no seasonal variation in water quality at the spring water because EC had an arithmetic mean (±SD) of 314.5 ± 41.4 μS cm^−1^ with a CV of 13%, and pH was 6.5 ± 0.4 with a CV of 6%. EC increases with increasing water temperature, which is reported by Hanya and Ogura [50]. Therefore, as changes in water temperature were small, the changes in EC were also considered to be small. However, the scope of this study is limited by the measurements made (outlined in Table 2) and as a result of the lack of detailed information on the depths of the spring water sources as well as their geological and chemical compositions. Obtaining these details in the future may provide additional information. In particular, measuring chemical composition such as ion concentrations and total dissolved solids, which are known to influence the behavior of ^226^Ra [2,17], the parent nuclide of ^222^Rn, in water could provide a better indication of the behavior of these radionuclides in groundwater sources.

The conservative annual effective ingestion dose for ^222^Rn and ^226^Ra obtained in this study of 8 μSv is significantly smaller than the estimated overall annual effective dose of 2.2 mSv from natural radiation (=2.2 mSv) to the Japanese population [41]. The WHO has adopted a pragmatic and conservative approach with an individual dose criterion of 0.1 mSv for the annual consumption of drinking water, regardless of the origin of radionuclides [13]. The evaluated annual effective dose accounts for 8% of this WHO criterion. It is, therefore, important to carefully investigate other radionuclides as well in the future.

## 5. Conclusions

This article described the results of measurements of ^222^Rn and ^226^Ra concentrations in spring water, in Hirosaki City, Aomori Prefecture, located in the northern part of Honshu Island in the Japanese archipelago. Spring water samples were collected from August 2016 to September 2016, at 15 locations that are known drinking water supplies and where sampling was possible. In addition, at one of these sites (Site No. 15), spring water samples were collected once a week from June 2016 to June 2017, to observe the seasonal variation of radon concentration. Results indicate that the measured ^222^Rn concentrations (=12.7 ± 6.1 Bq L^−1^) and ^226^Ra concentrations (<0.019–0.022 Bq L^−1^) did not exceed the reference values of other international organizations. In addition, the results of periodic measurements at Site No. 15 demonstrated no seasonal variation in ^222^Rn concentrations throughout the year. Finally, the conservative annual effective ingestion dose for ^222^Rn and ^226^Ra obtained in this study (8 μSv) is smaller than the Japanese population dose arising from natural radiation. However, this dosage accounts for 8% of the World Health Organization’s individual dosing criterion of 0.1 mSv. Therefore, the authors suggest that the contribution from other radionuclides be evaluated as well in the future.

## Figures and Tables

**Table 1:** The basement geology and sampling dates of the 15 sampling sites.

Site No.	Basement Geology	Sampling Date
1	Alluvium	4 August 2016
2	Dacite–Andesite Lava (after the Pliocene)	17 August 2016
3	Dacite–Andesite Lava (after the Pliocene)	18 August 2016
4	Alluvium	21 August 2016
5	Dacite–Andesite Lava (after the Pliocene)	22 August 2016
6	Dacite–Andesite Lava (after the Pliocene)	25 August 2016
7	Andesite Lava/Pyroclastic Rock (middle-upper Miocene)	29 August 2016
8	Terrace deposit	5 September 2016
9	Alluvium	7 September 2016
10	Terrace Deposit	8 September 2016
11	Pyroclastic Rock (after middle Pleistocene)	15 September 2016
12	Pyroclastic Rock (after middle Pleistocene)	16 September 2016
13	Pyroclastic Rock (after middle Pleistocene)	19 September 2016
14	Andesite Lava/Pyroclastic Rock (middle-upper Miocene)	26 September 2016
15	Alluvium	17 June 2016–21 June 2017

**Table 2:** Measurement results in the 15 sampling sites.

Site No.	pH	EC (μS cm^−^^1^)	Water Temp. (°C)	Atm. Temp. (°C)	^222^Rn Conc. ^a^ (Bq L^−1^)	^226^Ra Conc. ^b^ (Bq L^−1^)
1	6.5	250	15.2	27.5	16.5 ± 1.0	<MDC (0.0096)
2	7.2	153	14.7	14.7	6.7 ± 0.2	<MDC (0.0080)
3	6.4	86	19.7	24.9	13.9 ± 0.9	0.0098 ± 0.0031
4	6.2	111	21.7	25.7	11.5 ± 0.7	<MDC (0.012)
5	6.7	89	7.4	22.5	5.3 ± 0.5	<MDC (0.0089)
6	6.5	100	6.9	24.4	6.3 ± 1.0	<MDC (0.0085)
7	5.7	220	12.5	28.0	16.6 ± 0.6	<MDC (0.0099)
8	6.6	119	9.9	22.1	11.0 ± 0.8	<MDC (0.0099)
9	6.6	68	9.7	20.9	26.7 ± 1.1	0.0093 ± 0.0030
10	6.1	185	11.3	20.1	8.0 ± 0.7	<MDC (0.0095)
11	5.8	140	10.3	22.1	18.8 ± 0.8	0.022 ± 0.0071
12	6.2	198	9.7	20.6	18.6 ± 1.2	<MDC (0.0097)
13	5.9	164	9.9	17.8	7.9 ± 0.3	<MDC (0.0082)
14	6.2	240	10.2	19.1	7.7 ± 1.0	<MDC (0.0096)
15 ^c^	5.5–7.2	147–370	8.8–16.5	−4.3–33.4	12.2–18.6 (15.3 ± 1.2)	<MDC (0.019) ^d^

^a^ Measured values and uncertainties (*k =* 1) are indicated (Site No. 1–14). In Site No. 15, the range during the measurement period is indicated, and the arithmetic mean and SD are indicated in parentheses. ^b^ Measured values and uncertainties (*k =* 1) or MDCs are indicated. ^c^ The data quantity for measurement items’ expected ^226^Ra concentration is 50. ^226^Ra concentration is the result from a sample (sampling date: 10 September 2016). ^d^ This MDC, which is about twice as high as MDCs at other sites, results from its relatively low yield (=47%).

**Table 3:** The annual effective ingestion dose for ^222^Rn and ^226^Ra.

Site No.	The Annual Effective Dose (μSv)		
	^222^Rn	^226^Ra	Total
1	8 ± 0.5	<1	<10
2	3 ± 0.1	<1	<5
3	7 ± 0.5	1 ± 0.3	8 ± 1
4	6 ± 0.4	<2	<7
5	3 ± 0.2	<1	<4
6	3 ± 0.5	<1	<4
7	8 ± 0.3	<1	<10
8	6 ± 0.4	<1	<7
9	13 ± 0.5	1 ± 0.3	14 ± 1
10	4 ± 0.3	<1	<5
11	9 ± 0.4	2 ± 0.7	12 ± 1
12	9 ± 0.6	<1	<11
13	4 ± 0.2	<1	<5
14	4 ± 0.5	<1	<5
15	8 ± 0.005	<2	<10

**Table 4:** A comparison of ^222^Rn concentrations in drinking water.

Country	Location	Description	^222^Rn Conc. (Bq L^−1^)	Ref.
Japan	35 sites of “Meisui-100-sen”	Spring water	0.24–98.91 Ave. = 12.98	[30]
	Wakasa area, Fukui	Tap water	1.2–104 Median = 11.2	[31]
	Rokko area, Hyogo	Well water	2.6–78.6	[16]
	Ningyo-Toge area, Okayama and Tottori	Tap/well/spring water	0.1–230	[32]
Russia	Ural	Drinking water	57–92	[33]
Serbia	Niska Banja	Drinking water	430 ± 46	[33]
Spain	Catalonia	Groundwater	1.4–104.9	[7]
Germany	–	Drinking water	<1.3–1800	[34]
China	Beijing	Public water	<0.268–29.00	[35]
		Well water	1.45–49.00	[35]
Japan	Hirosaki, Aomori	Spring water	5.3–26.7 Ave. ± SD = 12.7 ± 6.1	This study

## Data Availability

Not applicable.
